# Exploring the role of autistic traits in treatment-resistant and clozapine-resistant schizophrenia: a comparative study

**DOI:** 10.3389/fpsyt.2025.1541469

**Published:** 2025-04-29

**Authors:** Ahmet Selim Başaran, Nur Nihal Türkel, Buket Koparal, Aslı Kuruoğlu

**Affiliations:** ^1^ Unye State Hospital, Ordu, Türkiye; ^2^ Penitentiary Campus State Hospital, Ankara, Türkiye; ^3^ Faculty of Medicine, Gazi University, Ankara, Türkiye

**Keywords:** schizophrenia, treatment-resistant schizophrenia, clozapine-resistant schizophrenia, autism spectrum disorder, autistic traits

## Abstract

**Background:**

Treatment resistance in schizophrenia is a major clinical challenge. While autistic traits are often more pronounced in patients with treatment-resistant schizophrenia (TRS), limited data exist on clozapine-resistant schizophrenia (CRS). This study aims to explore the relationship between autistic traits and treatment resistance in schizophrenia, with a focus on both TRS and CRS and to evaluate whether these traits could predict treatment outcomes.

**Methods:**

A total of 86 patients were included, divided into three groups: non-treatment-resistant schizophrenia (NRS, n=37), treatment-resistant schizophrenia (TRS, n=26), and clozapine-resistant schizophrenia (CRS, n=23). Psychopathology was assessed using the Positive and Negative Syndrome Scale (PANSS), while autistic traits were measured with the PANSS Autism Severity Scale (PAUSS) and the Autism Spectrum Quotient (AQ). Multinomial logistic regression models were used to determine the predictive value of autistic traits for TRS and CRS.

**Results:**

Statistically significant differences were identified between the groups in PAUSS (p<0.001) and AQ (p<0.001) scores, indicating variations in autistic traits. PAUSS scores were predictive of TRS and CRS relative to NRS but did not differ between TRS and CRS. In contrast, AQ scores showed significant differences between TRS and CRS. Both PAUSS and AQ were negatively correlated with functionality as measured by the Global Assessment of Functioning, highlighting the impact of autistic traits on daily functioning.

**Conclusions:**

The results indicate that autistic traits are associated with resistance to treatment, as PAUSS scores are predictive of the development of treatment-resistant and clozapine-resistant schizophrenia. However, the lack of statistically significant differences between TRS and CRS in PAUSS scores suggests that clozapine resistance may be influenced by additional factors beyond the autistic traits measured by PAUSS. To better understand the relationship between clozapine resistance and autistic traits, future research should not only focus on the autistic traits captured by PAUSS but also consider broader autism phenotypes or other distinct psychopathological processes. Such studies could offer deeper insights into the complex mechanisms that drive clozapine resistance and help identify new paths for treatment and intervention.

## 1 Introduction

Schizophrenia is a severe and chronic psychiatric disorder characterized by a wide range of symptoms, including positive symptoms (hallucinations and delusions), negative symptoms (such as social withdrawal and lack of motivation), and cognitive impairments (1). Despite significant advancements in the pharmacological treatment of schizophrenia, a considerable number of patients exhibit inadequate response to standard antipsychotic treatments (2–5). This phenomenon is referred to as treatment-resistant schizophrenia (TRS). It is defined as the lack of significant improvement following at least two trials of different antipsychotic drugs at adequate doses for an appropriate duration, typically 6–12 weeks (6).

Clozapine, an atypical antipsychotic, is often considered the treatment of choice for patients with TRS (7). It is the only medication with proven efficacy in this population (8–10). However, approximately 30-60% of TRS patients do not respond adequately to clozapine treatment, leading to a subset of patients classified as clozapine-resistant schizophrenia (CRS) (6, 11). These patients remain a particularly challenging group to manage, as there are limited treatment options available beyond clozapine (6). The mechanisms underlying both TRS and CRS are poorly understood. Still, recent evidence suggests that neurodevelopmental factors, including autistic traits, may play a role in the development of treatment resistance (12, 13).

Autistic traits refer to subthreshold features of autism spectrum disorder (ASD) observed in individuals who do not meet the full diagnostic criteria for ASD (14). These traits may lead to difficulties in social interaction, communication, and repetitive behaviors, and have been observed to be more common in psychiatric populations, such as individuals with schizophrenia (15–18). The relationship between schizophrenia and autism has been debated for over a century. Early psychiatrists, such as Eugen Bleuler, used the term “autism” to describe the withdrawal from reality observed in schizophrenia (19, 20). Although the disorders are now considered distinct entities, there is growing evidence that they share certain neurobiological and developmental pathways, as well as overlapping clinical features (21–23).

Several studies have explored the impact of autistic traits on schizophrenia. The findings suggest that individuals with higher levels of autistic traits may experience poorer social functioning, more severe negative symptoms, and a diminished response to antipsychotic treatment (18, 24, 25). Neurodevelopmental theories suggest that both schizophrenia and autism spectrum disorders may share early developmental disruptions involving social cognition and synaptic pruning (24–26). These disruptions can lead to persistent negative symptoms and poor response to dopaminergic treatments, which is central to TRS/CRS. However, most research has focused on the general population of schizophrenia patients, with few studies explicitly examining the role of autistic traits in TRS. This study aims to fill this gap by comparing autistic traits in non-treatment-resistant (NRS), treatment-resistant (TRS), and clozapine-resistant (CRS) schizophrenia patients and assessing how these traits can predict treatment resistance.

The hypothesis of the current study is twofold: (1) patients with TRS and CRS will exhibit higher levels of autistic traits compared to those with NRS, and (2) autistic traits, as measured by the PAUSS, will predict treatment resistance, with more severe traits associated with both TRS and CRS. By exploring these questions, we aim to gain a better understanding of the role of autistic traits in the pathophysiology of treatment resistance in schizophrenia.

## 2 Materials and methods

### 2.1 Participants and procedure

The study sample consisted of patients who presented to the Outpatient Psychiatry Clinic at Gazi University Hospital between September 2023 and February 2024, with a diagnosis of schizophrenia confirmed according to DSM-5 criteria. The inclusion criteria were as follows: (1) age between 18 and 65 years, (2) a confirmed DSM-5 diagnosis of schizophrenia, (3) absence of any other psychiatric or neurological comorbidities, especially autism spectrum disorder (ASD), intellectual disability, or dementia, (4) no history of alcohol or substance use disorder, and (5) no recent history of psychotic exacerbations, changes in medication, or hospitalization within the previous six months. These criteria were applied to ensure that the sample was homogenous in terms of schizophrenia diagnosis and to focus on subclinical autistic traits rather than a comorbid ASD diagnosis, which could confound or overshadow schizophrenia-related features.

Initially, semi-structured interviews were conducted with 98 patients who met the study’s inclusion criteria. These interviews were carried out prior to collecting data on the patients’ antipsychotic treatment histories to avoid bias related to prior knowledge of study hypotheses. Following the Positive and Negative Syndrome Scale (PANSS) assessments, four patients were excluded from the study as they did not meet the predefined remission criteria, which were based on the criteria set by The Remission in Schizophrenia Working Group (27). Based on these criteria, remission requires a score of three or less on each of the PANSS items P1, P2, P3, and G9. The criteria of the Remission in Schizophrenia Working Group also include the items N1, N4, N6, and G5; however, since these items are part of the PANSS Autism Severity Scale (PAUSS) scoring system, they were excluded from the remission criteria in this study to avoid any confounding in the measurement of autistic traits.

The research team conducted a thorough review of the remaining 94 patients’ medical histories and antipsychotic treatment records. Five additional patients were excluded due to insufficient data to evaluate their treatment history, leaving 89 patients for further analysis. These patients were subsequently administered the Autism Spectrum Quotient (AQ) to assess autistic traits further. Based on the information in the literature, patients who scored 32 or higher on the AQ or 30 or higher on the PAUSS were re-evaluated to determine if they could be on the autism spectrum, in order to rule out the likelihood of ASD. This additional assessment ensured that individuals with a high probability of ASD were appropriately considered in the study (28, 29). For patients exceeding this cutoff, their symptoms and developmental histories were reviewed in consultation with a psychiatrist experienced in ASD. As a result of this secondary evaluation, three patients for whom an ASD diagnosis could not be definitively ruled out were excluded from the study. Subsequently, the remaining 86 patients to be included in the analysis were divided into three groups: 37 in the NRS group, 26 in the TRS group, and 23 in the CRS group. The NRS group comprised patients who responded to antipsychotic treatment other than clozapine. The TRS group included patients who, despite receiving two different antipsychotic medications at doses equivalent to at least 600 mg/day of chlorpromazine over a 12-week period (with a minimum of six weeks at therapeutic doses), exhibited less than a 20% reduction in PANSS scores but later achieved remission with clozapine treatment (6). The CRS group consisted of patients who, despite receiving clozapine monotherapy at a minimum dose of 500 mg/day for at least three months or reaching a clozapine blood level of 350 ng/ml, showed less than a 20% reduction in PANSS scores and only achieved remission with the addition of other antipsychotic treatments (6). In the CRS group, the additional antipsychotics had to be administered at therapeutic doses and for adequate durations.

The study was approved by the Gazi University Ethics Committee, and all participants provided informed consent.

### 2.2 Measures

To assess psychopathology, the PANSS was administered to all patients. The PANSS is a 30-item, clinician-administered rating scale that measures the severity of positive symptoms (PANSS-T), negative symptoms (PANSS-N), and general psychopathological symptoms. Scores on the PANSS range from 30 to 210, with higher scores indicating greater psychopathological severity (30).

Autistic traits were assessed using two different tools: the PAUSS and the AQ. The PAUSS is a semi-structured interview that was developed from items in the PANSS to specifically measure autistic traits in schizophrenia patients (29). It focuses on three core dimensions of autism: social communication impairments, restricted interests, and repetitive behaviors. The PAUSS was validated against the Autism Diagnostic Observation Schedule and the AQ in both ASD and schizophrenia populations. The AQ is a 50-item self-report questionnaire designed to measure autistic traits in the general population. Higher AQ scores indicate more severe autistic traits (28).

Functionality was assessed using the Global Assessment of Functioning (GAF) scale, which provides a numerical rating of overall psychological, social, and occupational functioning. GAF scores range from 0 to 100, with higher scores indicating better functioning (31).

### 2.3 Statistical analyses

Statistical analyses were conducted using SPSS 29.0 software. Descriptive statistics were used to summarize sociodemographic and clinical characteristics. One-way ANOVA was used to compare continuous variables between the NRS, TRS, and CRS groups, as these variables followed a normal distribution. To control for the potential influence of positive symptom severity, an ANCOVA was conducted to compare AQ scores across groups, with PANSS-Positive included as a covariate. Chi-square tests were applied for categorical variables. Multinomial logistic regression analyses were performed to assess the predictive value of autistic traits on treatment resistance, with PAUSS scores entered as independent variables and NRS, TRS, and CRS group membership as the dependent variables.

Additionally, Pearson correlation analyses were used to examine the relationships between PANSS, PAUSS, AQ, and GAF scores. Linear regression analysis was applied to assess the impact of autistic traits on functionality, with GAF scores as the dependent variable and PAUSS and AQ scores as independent variables. The level of statistical significance was set at p<0.05 for all analyses.

## 3 Results

### 3.1 Sociodemographic and clinical characteristics

**Table 1** presents the demographic and clinical features of the participants in the non-treatment-resistant (NRS), treatment-resistant (TRS), and clozapine-resistant (CRS) groups. While there were no significant differences in age or gender distribution across groups (p > 0.05), the number of prior hospitalizations was significantly higher in the CRS group compared to NRS (p < 0.001).

**Table 1:** Sociodemographic, clinical characteristics and, autistic trait scores of patients with non-treatment-resistant, treatment-resistant, and clozapine-resistant schizophrenia.

	NRS (n=37)	TRS (n=26)	CRS (n=23)	Analysis				
				ANOVA (F value)	ANOVA (p value)	*post-hoc* (P value)		
						NRS vs TRS	NRS vs CRS	TRS vs CRS
**Age**	45.45 (11.54)	50.34(8.43)	45.39(7.98)	2.463	0.067	–	–	**-**
**Male Sex (n)**	24 (%64.9)	19 (%65.4)	20 (%87)	–	–	–	–	–
**Age at Onset**	25.4 (7.28)	24.3 (5.12)	21.52 (4.74)	2.939	0.058	–	–	–
**Illness Duration (y)**	20.78 (9)	26.03 (7.91)	20.82 (7.97)	3.197	0.054	**-**	–	–
**Hospitalizations (n)**	1.81(1.76)	2.73(1.21)	3.78 (2.31)	8.657	**< 0.001**	–	**< 0.001**	
**PANSS-P**	9.78 (2.89)	9.88 (2.12)	12.91 (3.65)	9.415	**< 0.001**	–	**0.004**	**0.004**
**PANSS-N**	14.18 (6.48)	18.30 (5.75)	20.00 (6.44)	6.926	**0.002**	**0.036**	**0.002**	–
**PANSS-T**	46.08 (14.55)	50.26 (10.53)	58.73 (13.17)	6.630	**0.002**	**-**	**0.001**	**-**
**GAF**	80.16 (19.27)	75.38 (16.29)	59.43 (20.96)	8.776	**< 0.001**	**-**	**< 0.001**	**0.013**
**PAUSS**	15.32 (6.40)	20.92 (6.78)	23.17 (7.08)	11.061	**< 0.001**	**0.005**	**< 0.001**	**-**
AQ*	18.75 (5.11)	23.03 (5.63)	27.17 (4.96)	11.483	**< 0.001**	**0.008**	**0.001**	**0.028**

Data are mean (SD). Bold values indicate statistically significant group differences (p < 0.05). ANOVA, analysis of variance; PANSS, Positive and Negative Syndrome Scale; PANSS-P, PANSS Positive Subscale; PANSS-N, PANSS Negative Subscale; PANSS-T, Total PANSS score calculated as the sum of all subscales; PAUSS, PANSS Autism Severity Scoring; AQ,Autism Spectrum Quotient; GAF, Global Assessment of Functioning Scale; NRS, Non-treatment-resistant Schizophrenia; TRS, Treatment-resistant Schizophrenia; CRS, Clozapine-resistant Schizophrenia; y, year.AQ scores were compared across groups using ANCOVA, with PANSS-P included as a covariate.

### 3.2 Psychopathology and functionality

**Table 1** summarizes the comparisons of psychopathology measures (PANSS subscales and total scores) and functionality (GAF) among the three groups. Notably, both the CRS and TRS groups had higher PANSS negative (PANSS-N) scores than the NRS group. Although the TRS group’s negative symptoms also differed significantly from NRS (p = 0.036), overall severity was most pronounced in CRS. In line with this, CRS patients demonstrated the lowest GAF scores (p < 0.001), suggesting poorer overall functioning despite being in clinical remission.

### 3.3 Autistic traits

As shown in **Table 1**, autistic trait measures (AQ, PAUSS) differed significantly among the groups. Both TRS and CRS patients exhibited higher AQ and PAUSS scores compared to the NRS group (p < 0.01). Although PAUSS did not distinguish between TRS and CRS, AQ scores were significantly higher in the CRS group than in TRS (p = 0.022). This pattern suggests that broader autistic phenotypes (captured by AQ) might be more relevant for understanding clozapine resistance.

### 3.4 Correlations and regression models

Correlation analyses demonstrated significant positive correlations between AQ scores and PANSS-P, PANSS-N, and PANSS-T scores (r=0.510, r=0.593, and r=0.611, respectively), indicating that more severe autistic traits were associated with more severe psychopathology (**Table 2**). Similarly, PAUSS scores were positively correlated with all PANSS subscales, suggesting that autistic traits are closely linked to overall symptom severity in schizophrenia.

**Table 2:** Correlations between autistic traits (PAUSS and AQ), psychopathology (PANSS), and functionality (GAF) scores.

	PAUSS	AQ
**PANSS-P**	r= 0.557*	r= 0.510*
**PANSS-N**	r= 0.936*	r= 0.593*
**PANSS-T**	r= 0.831*	r= 0.611*
**GAF**	r= -0.753*	r= -0.635*
**PAUSS**	r=1.000	r= 0.647*
**AQ**	r= 0.647*	r=1.000

Data are Pearson correlation coefficients (r) between autistic traits, psychopathology, and functioning measures.Bold values indicate statistically significant correlations (p < 0.001). PAUSS, PANSS Autism Severity Scoring (derived from the Positive and Negative Syndrome Scale); PANSS, Positive and Negative Syndrome Scale; PANSS-P, Positive Symptom Subscale; PANSS-N, Negative Symptom Subscale; PANSS-T, Total PANSS score (sum of positive, negative, and general subscales); AQ, Autism Spectrum Quotient; GAF, Global Assessment of Functioning Scale.

Linear regression analysis revealed that both PAUSS and AQ scores were significant predictors of GAF scores, with higher autistic traits associated with lower functionality (F=63.448, p<0.001) (**Table 3**). The multinomial logistic regression model showed that an increase in PAUSS scores predicted the development of treatment resistance and clozapine resistance in patients with schizophrenia (**Table 4**). Specifically, higher AQ scores and earlier age at onset emerged as predictors for CRS (p<0.001), underscoring a potential neurodevelopmental underpinning in this subgroup.

**Table 3:** Regression analysis of AQ and PAUSS scores with GAF score.

	B	S.E.	β	t	p	OR (%95 CI)	
						LB	UB
**Constants**	122.879	5.289		23.232	**< 0.001**	112.359	133.399
**AQ**	-0.837	0.298	-0.254	-2.806	**0.006**	-1.431	-0.244
**PAUSS**	-1.623	0.248	-0.589	-6.506	**< 0.001**	-2.119	-1.127

Data are unstandardized coefficients (B), standard errors (S.E.), standardized coefficients (β), t values, and significance levels. Bold values indicate statistically significant results (p < 0.05). PAUSS, Positive and Negative Syndrome Scale Autism Severity Scoring; AQ, Autism Spectrum Quotient; GAF, Global Assessment of Functioning Scale.

**Table 4:** Multinomial logistic regression model predicting treatment-resistance and clozapine-resistance based on autistic traits and clinical variables.

	X^2^	p	TRS				CRS			
			B(SE)	Exp(B)	Wald	p	B(SE)	Exp(B)	Wald	p
**PAUSS**	17.272	**< 0.001**	0.131(0.048)	1.140	7.516	**0.006**	0.199(0.057)	1.220	12.302	**< 0.001**
**Age at Onset**	10.959	**0.004**	-0.037(0.051)	0.964	0.521	0.470	-0.211(0.078)	0.810	7.356	**< 0.001**
**Ilness Duration (y)**	11.002	**0.004**	0.065(0.040)	1.067	2.582	0.108	-0.075(0.046)	0.928	2.679	0.102
**Hospitalizations (n)**	14.954	**0.001**	0.268(0.200)	1.307	1.792	0.181	0.861(0.255)	1.435	11.384	**0.001**

Data are regression coefficients (B), standard errors (SE), odds ratios (Exp(B)),Wald statistics, and p values. Bold values indicate statistically significant predictors (p < 0.05). PAUSS, Positive and Negative Syndrome Scale Autism Severity Scoring; TRS, Treatment-resistant Schizophrenia; CRS, Clozapine-resistant Schizophrenia; y, year.

## 4 Discussion

This study provides new insights into the impact of autistic traits on both treatment-resistant schizophrenia (TRS) and clozapine-resistant schizophrenia (CRS). The findings demonstrate that autistic traits are more prominent in patients with treatment-resistant forms of schizophrenia (TRS and CRS) compared to those who respond to non-clozapine antipsychotic treatments (NRS). Furthermore, autistic traits, as measured by the PAUSS, were found to be a predictor of the development of both TRS and CRS, indicating the potential significance of these traits in understanding and managing treatment resistance in schizophrenia.

Several clinical features linked to TRS have been identified in the literature. However, many studies focus primarily on predictors of treatment nonresponse without directly comparing patients with treatment resistance to those without it. Some studies have suggested that an earlier age of onset is associated with treatment resistance (32–34). In our study, while no significant difference in age of onset was observed between groups, a younger age of onset was found to predict clozapine resistance. This finding is partially consistent with the existing literature that supports the association between early onset and treatment resistance; however, it is difficult to compare directly with most studies, as they do not distinguish between clozapine-resistant (CRS) and treatment-resistant (TRS) patients as separate groups. Additionally, no significant gender differences were found between the groups, consistent with findings from the cohort study by Wimberley and colleagues (35).

All patients included in the study were assessed as being in clinical remission. However, significant differences were observed between the groups in PANSS-P, PANSS-N, and PANSS-T scores. These findings suggest that treatment-resistant patients, particularly those resistant to clozapine, exhibit more severe psychopathology compared to non-treatment-resistant patients, even when they are clinically stable. Additionally, the positive correlations between PANSS, PAUSS, and AQ scores highlight the association between autistic traits and greater psychopathological severity. These results align with previous studies that have shown autistic traits in individuals with schizophrenia are associated with a reduced response to antipsychotic treatment (18, 24, 25).

One of the key findings of this study is the difference in AQ scores between the TRS and CRS groups, which suggests that broader autism phenotypes may play a role in clozapine resistance. The AQ was initially developed for use in the general population, and higher scores may reflect a broader autism phenotype that captures subthreshold traits of autism that are not necessarily pathological but could influence treatment outcomes in schizophrenia (23). The lack of a significant difference in PAUSS scores between TRS and CRS patients raises important questions about the specific nature of clozapine resistance. While PAUSS was developed to measure autistic traits in schizophrenia, its overlap with negative symptoms could limit its ability to distinguish between different subtypes of treatment resistance. This overlap suggests that autistic traits measured by PAUSS in schizophrenia may be more closely related to the severity of negative symptoms and overall psychopathology rather than being a distinct feature that predicts clozapine resistance. This discrepancy also underscores a potential neurodevelopmental continuum, suggesting that clozapine-resistant patients may exhibit a distinct profile of autism-like traits that extends beyond the negative symptom spectrum traditionally measured by PAUSS. Future research should further delineate whether these broader traits reflect a separate underlying mechanism—possibly involving atypical neurodevelopmental pathways—or whether they represent a more severe form of negative symptomatology. Such clarification could refine our understanding of the “subtypes” within treatment-resistant schizophrenia and guide the development of more targeted interventions.

This study also highlights the importance of considering functionality in the assessment of autistic traits. Both PAUSS and AQ scores were negatively correlated with GAF scores, indicating that higher levels of autistic traits are associated with poorer social and occupational functioning. This finding is consistent with previous studies showing that autistic traits in schizophrenia are linked to poorer functional outcomes (18). Given the strong association between autistic traits and reduced functionality, clinicians should consider incorporating assessments of these traits into the treatment planning process for schizophrenia patients, particularly those with treatment resistance.

The results of this study have several important clinical implications. First, the findings suggest that autistic traits may be a useful predictor of treatment resistance in schizophrenia, particularly in distinguishing between NRS and TRS/CRS patients. Assessing autistic traits using tools like PAUSS and AQ could help clinicians identify patients who are more likely to develop treatment resistance, allowing for earlier intervention and potentially more tailored treatment approaches. Second, the difference in AQ scores between TRS and CRS patients indicates that broader autism phenotypes may be relevant in understanding clozapine resistance. This finding suggests that CRS may involve additional neurodevelopmental or psychopathological factors that autistic traits measured by PAUSS do not capture. Further research is needed to explore these factors, which could include genetic, neurobiological, or environmental influences. Third, the strong relationship between autistic traits and functionality highlights the need for comprehensive treatment plans that address both symptom severity and social functioning. Antipsychotic optimization alone may not suffice for patients who exhibit more pronounced social communication difficulties and rigid behavior patterns. In clinical practice, incorporating psychosocial interventions that target social cognition, such as social skills training or cognitive-behavioral therapy, could help ameliorate these autistic-like features and potentially enhance overall treatment response. By addressing these nuanced deficits, clinicians might improve both the quality of life and functional outcomes of patients who are less responsive to conventional medication management (36, 37).

While this study provides important insights into the relationship between autistic traits and treatment resistance in schizophrenia, several limitations should be noted. First, the cross-sectional design of the study limits our ability to draw conclusions about causality. Longitudinal studies are needed to determine whether autistic traits precede the development of treatment resistance or whether they emerge as a result of treatment failure. Second, the sample size was relatively small, particularly for the CRS group. Although the statistical analyses were adequately powered, larger samples would provide more robust estimates of the relationships between autistic traits and treatment resistance. Future studies should aim to replicate these findings in more extensive and more diverse populations. Third, the reliance on self-report measures like the AQ may introduce bias, particularly in a population with schizophrenia, where insight into symptoms may be limited. Although the study attempted to mitigate this limitation by including patients in remission, future studies should consider using additional clinician-administered tools to assess autistic traits. Finally, the study did not account for the potential effects of medication on autistic traits. Antipsychotic medications can have a wide range of effects on cognitive and social functioning, and it is possible that some of the observed differences in autistic traits between groups were influenced by differences in medication use. Future studies should control for medication type and dosage to isolate the effects of autistic traits on treatment resistance.

## 5 Conclusion

This study is the first to directly compare autistic traits among non-treatment-resistant (NRS), treatment-resistant (TRS), and clozapine-resistant (CRS) schizophrenia patients, offering new insights into how subthreshold autism spectrum features may influence clinical outcomes. The results indicate that autistic traits are significantly linked to treatment resistance, with PAUSS scores predicting both TRS and CRS status. Moreover, the observation that CRS patients demonstrate distinctly elevated AQ scores—beyond those seen in TRS—suggests that broader autism phenotypes could be a key factor differentiating this subgroup. Such a finding implies that CRS may involve additional neurodevelopmental mechanisms beyond those typically associated with TRS. Nevertheless, the identification of younger age at onset and a greater number of hospitalizations as predictors of CRS underscores the multifactorial nature of treatment resistance, highlighting that while autistic traits are crucial, other clinical factors further shape clozapine resistance trajectories.

Although the present study provides cross-sectional evidence linking autistic traits to different forms of treatment resistance, longitudinal designs are needed to determine whether these traits precede and predict the development of TRS or CRS, or whether they are exacerbated by prolonged illness course and multiple treatment failures. Prospective cohorts could clarify whether early identification of heightened autistic traits—especially social interaction difficulties—could serve as a marker for subsequent treatment refractoriness.

Overall, while this study shows that autistic traits play a prominent role in driving treatment resistance, the multilayered etiology of clozapine resistance demands a broad-based approach in both research and clinical management. Addressing not only neurodevelopmental and social-cognitive deficits but also the timing of illness onset, hospitalization history, and potentially relevant genetic or environmental factors will be essential for optimizing interventions and ultimately improving prognoses in these challenging patient populations.

## Data Availability

The original contributions presented in the study are included in the article/**Supplementary Material**. Further inquiries can be directed to the corresponding author/s.
