# Supplementary material for: Exploring the role of autistic traits in treatment-resistant and clozapine-resistant schizophrenia: a comparative study
Source: Front Psychiatry. 2025 Apr 29;16:1541469. doi: 10.3389/fpsyt.2025.1541469 (PMC12069336; doi:10.3389/fpsyt.2025.1541469)
Supplement: Supplementary file 1 [file Table1.docx]

**Supplementary Table.** The antipsychotics used by the patients included in the study and their average doses.

| **Antipsychotics** | **NRS**  **(n=37)** | | **TRS**  **(n=26)** | | **CRS**  **(n=23)** | |
| --- | --- | --- | --- | --- | --- | --- |
|  | **n** | **mg** | **n** | **mg** | **n** | **mg** |
| Amisulpiride | 10 | 860 | 0 |  | 6 | 733.33 |
| Aripiprazole | 6 | 18.75 | 0 |  | 7 | 26.43 |
| Clozapine | 0 |  | 26 | 465 | 23 | 636.46 |
| Flupenthixol Decanoate | 0 |  | 0 |  | 2 | 60 |
| Haloperidole | 0 |  | 0 |  | 2 | 10 |
| Olanzapine | 6 | 20.83 | 0 |  |  |  |
| Paliperidone Oral | 2 | 10.5 | 0 |  | 0 |  |
| Paliperidone Palmitate | 5 | 135 |  |  | 3 | 150 |
| Risperidone | 6 | 4.67 | 0 |  | 3 | 5.33 |
| Sulpiride | 2 | 1600 | 0 |  |  |  |
| Zuclopenthixol Decanoate | 0 |  | 0 |  | 3 | 400 |

The average doses of Flupentixol Decanoate, Paliperidone Palmitate, and Zuclopenthixol Decanoate are provided monthly, while the others are given daily.
